# Genome-Wide Analyses of the Temperature-Responsive Genetic Loci of the Pectinolytic Plant Pathogenic *Pectobacterium atrosepticum*

**DOI:** 10.3390/ijms22094839

**Published:** 2021-05-03

**Authors:** Natalia Kaczynska, Ewa Lojkowska, Magdalena Narajczyk, Robert Czajkowski

**Affiliations:** 1 Laboratory of Plant Protection and Biotechnology, Intercollegiate Faculty of Biotechnology, University of Gdansk and Medical University of Gdansk, Antoniego, Abrahama 58, 80-307 Gdansk, Poland; natalia.kaczynska@ug.edu.pl (N.K.); ewa.lojkowska@ug.edu.pl (E.L.); 2 Laboratory of Electron Microscopy, Faculty of Biology, University of Gdansk, Wita Stwosza 59, 80-308 Gdansk, Poland; magdalena.narajczyk@ug.edu.pl; 3 Laboratory of Biologically Active Compounds, Intercollegiate Faculty of Biotechnology, University of Gdansk and Medical University of Gdansk, Antoniego, Abrahama 58, 80-307 Gdansk, Poland

**Keywords:** *Erwinia atroseptica*, gene expression regulation, transposon, ecology, climate change

## Abstract

Temperature is one of the critical factors affecting gene expression in bacteria. Despite the general interest in the link between bacterial phenotypes and environmental temperature, little is known about temperature-dependent gene expression in plant pathogenic *Pectobacterium atrosepticum*, a causative agent of potato blackleg and tuber soft rot worldwide. In this study, twenty-nine *P. atrosepticum* SCRI1043 thermoregulated genes were identified using Tn5-based transposon mutagenesis coupled with an inducible promotorless *gusA* gene as a reporter. From the pool of 29 genes, 14 were up-regulated at 18 °C, whereas 15 other genes were up-regulated at 28 °C. Among the thermoregulated loci, genes involved in primary bacterial metabolism, membrane-related proteins, fitness-corresponding factors, and several hypothetical proteins were found. The Tn5 mutants were tested for their pathogenicity *in planta* and for features that are likely to remain important for the pathogen to succeed in the (plant) environment. Five Tn5 mutants expressed visible phenotypes differentiating these mutants from the phenotype of the SCRI1043 wild-type strain. The gene disruptions in the Tn5 transposon mutants caused alterations in bacterial generation time, ability to form a biofilm, production of lipopolysaccharides, and virulence on potato tuber slices. The consequences of environmental temperature on the ability of *P. atrosepticum* to cause disease symptoms in potato are discussed.

## 1. Introduction

Potato (*Solanum tuberosum* L.) is one of the main food crops worldwide. It is currently grown over an area estimated at 17 million hectares, with an annual yield estimated at 370 million tons [1]. Potato is generally a crop of temperate climates, but it is also grown in subtropical and tropical areas, demonstrating its adaptability to a wide range of environmental conditions. 

Among the most harmful and devastating bacterial diseases affecting worldwide potato production are potato blackleg of field-grown plants and tuber soft rot during storage and transit. Both diseases are caused by pectinolytic Soft Rot *Pectobacteriaceae* (SRP): *Pectobacterium* spp. and *Dickeya* spp. [2,3,4]. 

The genus *Pectobacterium* currently includes 19 species [5,6,7,8,9,10,11,12,13], and the genus *Dickeya* gathers 12 recognized species [14,15,16,17]. *Pectobacterium* and *Dickeya* species are listed in the top 10 most important bacterial plant pathogens in agriculture based on their economic impact [18]. 

In addition, SRP can colonize and infect a wide range of crops other than potato and also infects ornamental plants. These bacteria can be present in plant tissue in latent infection, not causing any disease symptoms on host crops. *Pectobacterium* spp. and *Dickeya* spp. are also isolated from weeds and wild plants, irrigation and surface waters, insects, as well as from contaminated agricultural tools and equipment [4,19,20,21,22,23,24]. 

The distribution of the SRP is determined by the biotic and abiotic conditions under which the bacteria can persist and infect plants in the environment. Temperature is considered as one of the most important factors affecting disease development caused by SRP [25,26]. *Pectobacterium* spp. and *Dickeya* spp. differ in their optimal growth temperatures. For example, it has been demonstrated that *P. atrosepticum* grows faster at lower air temperatures (<25 °C); the optimal growth temperature for *P. parmentieri* and *P. brasiliense in vitro* is near 30 °C, while the optimal temperature for *D. solani* is ca. 35 °C [19,26,27]. 

Until the end of the last century, *P. atrosepticum* and *D. dianthicola* were considered responsible for most potato blackleg infections in Europe [2,4,23,27,28,29]. Around the 2000s, *D. solani* has become an important cause of blackleg and soft rot in Europe [3,4,30,31]. This pathogen has spread across Europe very rapidly and has been detected on potato in most European countries [4,23,30,32,33]. However, since 2012, a shift from *D. solani* as the dominant blackleg causing the agent to *P. parmentieri* and *P. brasiliense* has been observed [23,34,35,36,37]. *P. atrosepticum* still remains the dominant species causing potato blackleg and soft rot in specific temperate regions, including the United Kingdom, Norway, and Canada [38,39,40,41]. Results from the seed potato survey between 2013 and 2015 in England, Wales, and Scotland indicate that *P. atrosepticum* constituted over 89% of all positive samples [41]. *P. atrosepticum* was also one of the most frequently detected *Pectobacterium* species detected during surveys 2015–2017 in Northern Ireland [42].

*P. atrosepticum* (formerly known as *Erwinia carotovora* subsp. *atroseptica*) is considered a narrow host range pathogen restricted mainly to potato [43]. Nevertheless, the disease symptoms caused by *P. atrosepticum* have also been reported in pepper [44] and sunflower [45], where it caused soft rot. A relatively narrow host range may suggest that *P. atrosepticum* has lost genes required for pathogenesis on other plants or has acquired genes that limit its host range [46,47]. *P. atrosepticum* is principally found in cooler temperate regions worldwide, causing symptoms at average temperatures below 25 °C [2,43]. In contrast to many of the other SRPs, it does not grow at temperatures above 36 °C [5,48]. 

The climate change associated with increasing average temperatures over the growing season may cause a temperature-induced shift in the distribution of *Dickeya* spp. and *Pectobacterium* spp. worldwide. Various studies have demonstrated the effect of temperature on species dominance [19,27,49,50,51]. It has been shown that temperature modifies which pathogen predominates if more than one SRP species is present inside a rotting seed tuber [2]. Temperature also controls the expression of virulence factors in plant pathogens during infections. In contrast to human and animal pathogens, in plant pathogenic bacteria, many virulence genes are induced at lower temperatures (16–24 °C) and repressed at 28 °C, although their optimal growth temperatures range from 25 to 30 °C [52,53,54,55,56]. For instance, in *P. atrosepticum*, pectate lyase activity was reported to be maximal at 15–17 °C [57] and reduced at 30.5 °C [58].

Research of the past decades has revealed the general interest in bacterial phenotypes associated with growth temperatures. However, only a few model systems for thermo-responsiveness in plant pathogens have been reported until now [59,60,61]. Understanding how SRP adapts to different temperatures and how climate change influences the epidemiology of the diseases they cause in Europe is fundamental in the agricultural and food industry.

The purpose of this study was to characterize these *P. atrosepticum* transcriptional units (genes/operons) encoding factors contributing to environmental fitness and *in planta* virulence that were expressed exclusively at one of the two chosen temperatures (18 or 28 °C). This strategy was employed to help explain the observed shift of SRP pathogens in Europe from *P. atrosepticum* (dominant potato blackleg and soft rot pathogen in the past) to new emerging potato pathogens, including *D. solani*. For that, we used a random mutagenesis approach for the *P. atrosepticum* strain SCRI1043 [62,63], using Tn5 with a promotorless *gusA* reporter gene [64]. This approach depends on the Tn5-*gusA* transposons that merge target operons or genes with the promotorless reporter *gusA*. Expression of the *gusA* reporter occurs only when the expression of the gene/operon carrying the Tn5 transposon is activated. To identify temperature-regulated transcriptional units, we applied a rigorous gene selection protocol in which all genes/operons that were expressed at both temperatures (18 and 28 °C) were removed from our further analyses in a preliminary step. This allowed us to disclose bacterial genes/operons expressed exclusively in one or the other temperature. Selected thermoregulated loci were sequenced and further characterized to get insights into the role of temperature in the ecology of *P. atrosepticum*.

## 2. Results

### 2.1. Transposon Mutagenesis and Visual Estimation of β-glucuronidase Activity

A total of 5775 mutants of *P. atrosepticum* wild-type strain SCRI1043 were obtained after mutagenesis and screened for thermoregulated phenotypes. The estimated frequency of mini-Tn5 transposon transfer from the donor *E. coli* S17-1 λ-*pir* to recipient *P. atrosepticum* SCRI1043 was ca. 10^−5^ cells/recipient. The *β*-glucuronidase activity of all generated mutants was compared visually in duplicates at low (18 °C) and high (28 °C) temperatures on a solid M9 minimal medium supplemented with X-Gluc. Colonies of the individual *P. atrosepticum* mutants exhibited varied levels of GUS activity. The majority of tested *P. atrosepticum* SRI1043 mutants (around 98%) showed no difference in their GUS activity at low and high temperatures. A total of 109 mutants out of 5775 appeared to be thermoregulated with higher GUS activity at either 18 or 28 °C (blue and dark blue colonies) and were thus chosen for quantitative GUS assays.

### 2.2. Quantitative Spectrophotometric and Fluorometric GUS Assay

The 109 *P. atrosepticum* transposon mutants, showing thermoresponsive GUS phenotypes, were further tested in two subsequent quantitative assays: spectrophotometric and fluorometric GUS assays. GUS activity was considered temperature-dependent when the ratio of absolute GUS values (expressed as U/µg protein) obtained by fluorometric assay from 18 and 28 °C cultures was more significant than 1.5 or less than −1.5. Out of the 109 mutants tested, 40 mutants demonstrated significant temperature-dependent GUS activity, indicating that the miniTn5 transposon has been inserted downstream from the thermoregulated promoter. From those, 20 transposon mutants showed an increased GUS activity at 18 °C, and 20 mutants exhibited a high GUS activity at 28 °C. Relative GUS activity of the mutants at 18 °C ranged from 0.6 to 250 U/µg protein and at 28 °C ranged from 0.5 to 170 U/µg protein. The level of temperature induction expressed as the GUS activities ratio at 18 °C versus 28 °C or *vice versa* varied between 1.5- and 5-fold.

### 2.3. Phenotypic Characterization of P. atrosepticum Transposon Mutants

To investigate the physiological effect of the mutations in the temperature-regulated genes, 40 selected transposon mutants of *P. atrosepticum* SCRI1043 showing at least 1.5-fold change in the relative GUS activity at 18 °C or 28 °C were compared with the wild-type *P. atrosepticum* SCRI1043 for differences in their phenotypic characteristics such as cell morphology, exoenzyme production, motility, biofilm formation, *in vitro* growth rate, and ability to rot potato tuber tissue.

In comparison with the wild-type *P. atrosepticum* SCRI1043, none of the 40 analyzed transposon mutants showed differences in cell morphology in TEM analysis (data not shown). Likewise, the transposon mutants did not show any significant difference from the parental strain in swimming motility at 28 °C. However, in mutant PbaTn5-B76, the swimming motility was reduced compared to the wild-type *P. atrosepticum* SCRI1043 grown at 22 °C.

The level of exoenzyme production of *P. atrosepticum* transposon mutants and SCRI1043 was assessed by picking bacterial colonies onto enzyme assay plates for pectate lyases, cellulases, and proteases. All indicator plates showed a statistically significant reduction in the amount of enzyme produced only by one mutant, PbaTn5-B76, compared with SCRI1043, as determined by halo size (Table S1).

**Figure 1:**
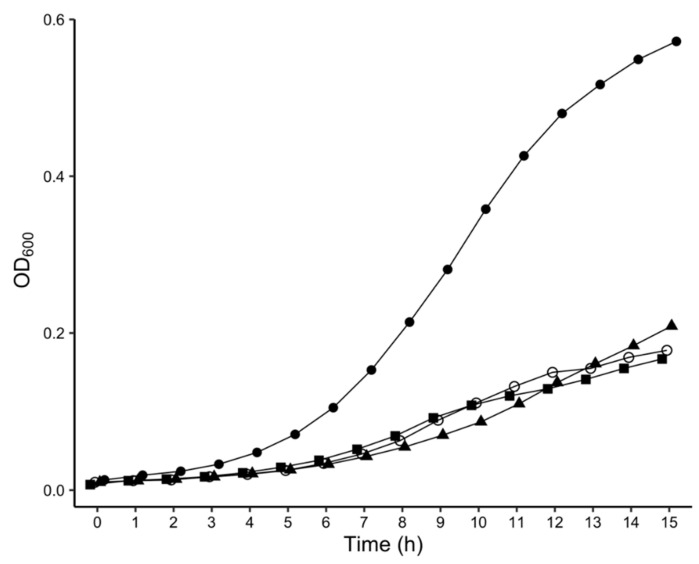
Growth curves of *P. atrosepticum* wild-type strain SCRI1043 (closed circle) and mutant strains PbaTn5-43 (closed square), PbaTn5-B58 (open circle), and PbaTn5-B76 (closed triangle) grown in M9 minimal media supplemented with 0.4% glycerol. The figure depicted only mutants where bacterial growth was significantly different from that of the wild-type *P. atrosepticum* SCRI1043. Bacterial growth was determined by measuring OD_600_ against a medium blank. The values are expressed as the mean (*n* = 6 from two independent experiments).

The growth curves of the 40 transposon mutants grown in M9 minimal medium supplemented with 0.4% glycerol as the sole carbon source were determined. The growth of the mutants PbaTn5-B76, PbaTn5-B58 and PbaTn5-43, was significantly slower than that of the wild-type SCRI1043 strain (Figure 1). The doubling times of PbaTn5-B76, PbaTn5-B58, PbaTn5-43 (1.9–2.0 h) were longer than the doubling time of SCRI1043 (1.4 h). The growth of the other 37 mutants was not significantly different from that of their parental SCRI1043 wild-type strain. 

**Figure 2:**
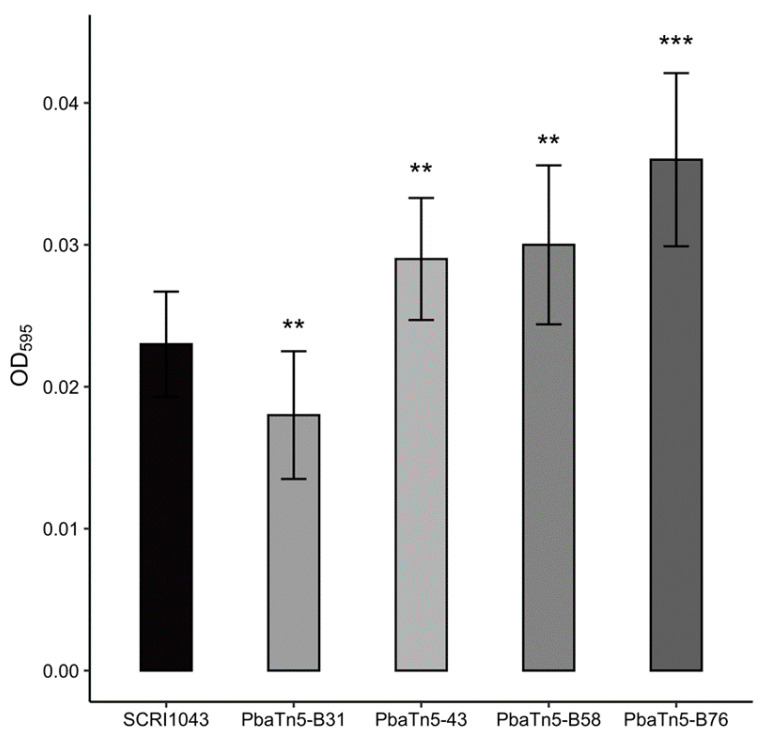
The *in vitro* biofilm formation ability of *P. atrosepticum* SCRI1043 wild-type strain and mutant strains differed in M9 minimal media supplemented with 0.4% glycerol. The figure depicted only mutants where the biofilm formation level was significantly different from the wild-type *P. atrosepticum* SCRI1043. All strains were examined in two independent experiments with duplicate samples, and the error bars indicate standard deviations. Statistically significant differences in biofilm formation between wild-type *P. atrosepticum* SCRI1043 and mutant strains are indicated (*** *p* < 0.001; ** *p* < 0.01) and were determined by the Student’s *t*-test.

The biofilm formation ability of transposon mutants was investigated using a crystal violet staining assay in polypropylene Eppendorf tubes. The results indicated that the *P. atrosepticum* SCRI1043 wild-type strain and all the mutant strains formed a biofilm on the inner surface of the polypropylene Eppendorf tubes. However, the level of biofilm formation capacity was relatively low. Three transposon mutants, PbaTn5-43, Pba-B58, and PbaTn5-B76, showed significantly enhanced biofilm formation compared to that of the wild-type SCRI1043 (*p* < 0.05) when the M9 minimal medium was supplemented with 0.4% glycerol (Figure 2). The biofilm formation ability of the mutant PbaTn5-B31, was significantly reduced compared to that of the wild-type strain SCRI1043.

### 2.4. Virulence of P. atrosepticum Tn5 Mutants

**Figure 3:**
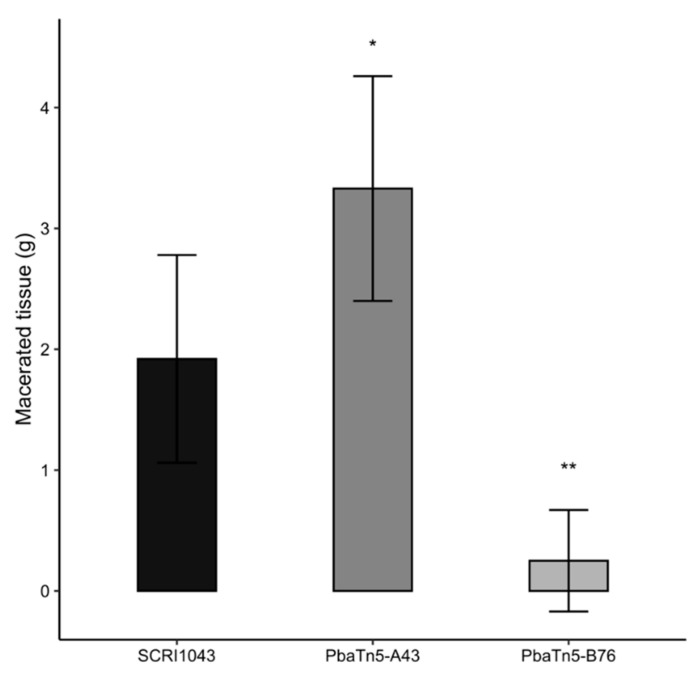
Maceration ability of the wild-type strain *P. atrosepticum* SCRI1043 and transposon mutant strains on potato tuber slices. The figure depicted only mutants where the level of potato tissue macerating ability was significantly different from that in wild-type *P. atrosepticum* SCRI1043. Bacteria (10^6^ per inoculation) of the wild-type strain and mutant strains were inoculated into wells on potato tuber slices. The maceration capacity of mutants (in grams of macerated tissue) was measured after 72 h of incubation at 28 °C. The error bars represent the SD of the *n* = 6 potato tuber slices. Asterisks indicate statistically significant differences in the degree of maceration of the mutants compared with the wild-type strain *P. atrosepticum* SCRI1043 (** *p* < 0.01; * *p* < 0.05), determined by the Student’s *t*-test.

To investigate the possible effect of transposon mutations on the virulence of *P. atrosepticum*, the ability of SCRI1043 and selected Tn5 transposon mutants to rot potato tuber tissue was investigated. The majority (95%) of the transposon mutants did not show any difference in tissue maceration capacity compared with the wild-type strain SCRI1043. The transposon mutant PbaTn5-B76 exhibited a significant decrease in the ability to macerate potato tuber tissue (85%, at *p* < 0.05) in comparison with the maceration ability of the wild-type strain SCRI1043 (Figure 3). In contrast, mutant PbaTn5-A43, showed a 50% elevated ability to macerate potato tubers than the one observed in the case of the wild-type strain SCRI1043.

The LPS profile of PbaTn5-B76 was investigated. LPS samples were analyzed by SDS gel electrophoresis. The PbaTn5-B76 mutant exhibited a truncated LPS as compared with the parental wild-type strain LPS. It is noteworthy that the total amount of LPS present in the PbaTn5-B76 appeared very much reduced (Figure S1A). Furthermore, the PbaTn5-B76 mutant showed weaker lactose fermentation when was plated on MacConkey agar compared to the wild-type strain SCRI1043 (Figure S1B).

### 2.5. Characterization of Transposon Insertion Sites

**Figure 4:**
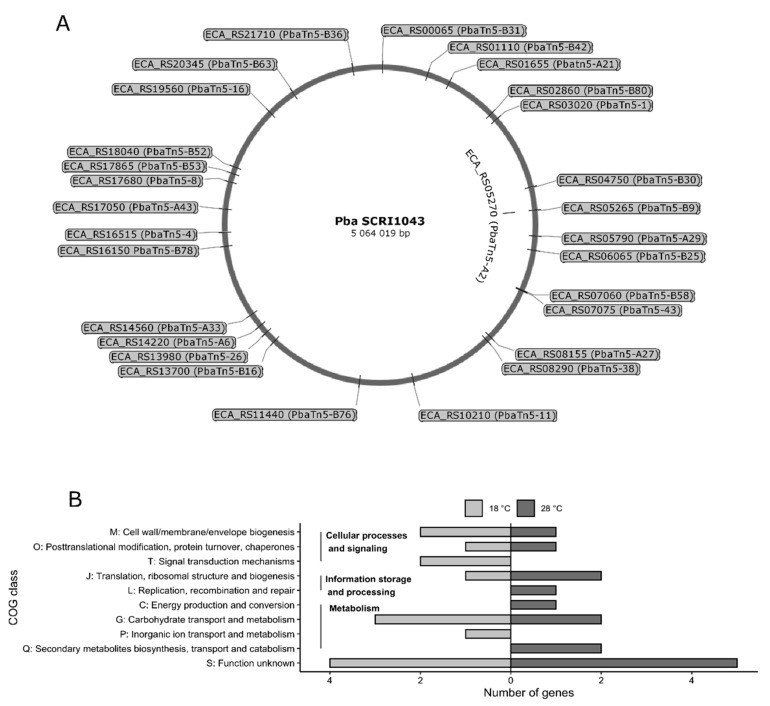
The distribution of the transposon insertions in the genome of *P. atrosepticum* strain SCRI1043. The diagram was drawn using *P. atrosepticum* SCRI1043 genome sequence information (GenBank: BX950851). Violet vertical lines represent the location of the transposon. The transposon mutant names (PbaTn5-x) are indicated. Gene names (ECA_RSx) according to the gene nomenclature for *P. atrosepticum* SCRI1043. The image was prepared using SnapGene^®^ software (**A**). Functional categories of twenty-nine thermoregulated loci based on clusters of orthologous groups (COG). Bar plot showing the number of genes under 10 different COG categories depicted on the *y*-axis according to four broad functional groups (**B**).

The transposon flanking regions for 29 transposon mutants of *P. atrosepticum* SCRI1043, which showed greater fold change of GUS activity or significant phenotypic difference from the parental strain, were sequenced and comparatively analyzed using BLAST against the sequenced *P. atrosepticum* genome strain SCRI1043 (accession no. BX950851) [63]. Analysis of the nucleotide sequences flanking the transposon insertion revealed that each mutant resulted from a unique insertion event. The insertions were in different loci throughout the genome of *P. atrosepticum* SCRI1043 (Figure 4A). Among the thermoregulated loci were those involved in bacterial metabolism (aminohydrolase, siderophore biosynthesis proteins, metallo-*β*-lactamase, transcriptional regulators), signal transduction mechanisms (sigma-E factor regulatory proteins), chaperons (chaperone protein ClpB), and also those involved in cell wall biogenesis (UDP-phosphate galactose phosphotransferase, UTP-glucose-1-phosphate uridylyltransferase) (Table 1). 

The proteins encoded by the 29 thermoregulated loci identified in this study were grouped into functional categories using the clusters of orthologous groups of proteins (COGs) from EggNOG database [65], as shown in Table 1. Of these, in mutants with increased GUS activity at 18 °C, five proteins were classified in Cellular processes and signaling related categories (O, M, T); four proteins were represented in Metabolism related categories (G, P); Information storage and processing related categories included one protein (J); Poorly characterized COG group S contained four proteins (Figure 4B). In mutants with increased GUS activity at 28 °C metabolism categories (C, G, Q) included five proteins; three proteins were classified in Information storage and processing related categories (J, L); two proteins were represented in Cellular processes and signaling categories (O, M); Poorly characterized COG group S contained five proteins (Figure 4B).

### 2.6. Time-Dependent Induction of Gene Expression Among P. atrosepticum Transposon Mutants

**Figure 5:**
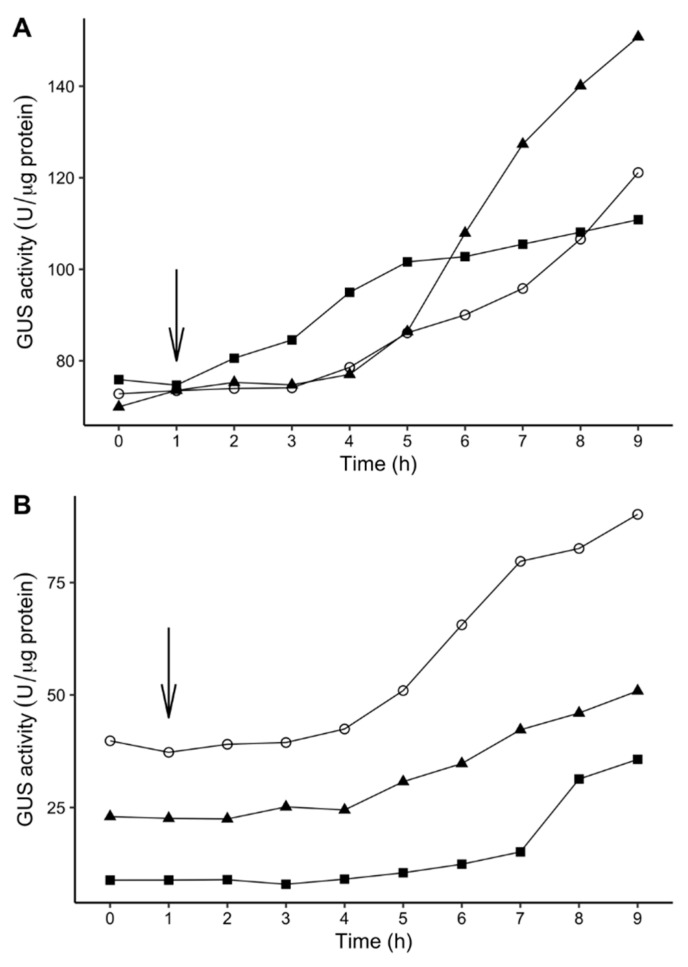
Effect of temperature shift on GUS activity in the transposon mutants of *P. atrosepticum* SCRI1043. Bacteria were grown to OD_600_ of 1.0 (t = 0 h) at the non-inductive temperature, incubated for an additional hour, and then shifted (arrow) to the *gusA*-expression-inductive temperature. Values are the means of three replicates per strain. Effect of temperature shift from 28 °C to 18 °C on GUS activity in *P. atrosepticum* SCRI1043 mutants with higher levels of GUS activity at 18 °C: PbaTn5-38 (open circle), PbaTn5-43 (closed triangle), PbaTn5-B76 (closed square) (**A**). Effect of temperature shift from 18 °C to 28 °C on GUS activity in *P. atrosepticum* SCRI1043 mutants with higher levels of GUS activity at 28 °C: PbaTn5-A33 (closed square), PbaTn5-B25 (open circle), PbaTn5-B42 (closed triangle) (**B**).

To investigate the time needed for induction of GUS activity in transposon mutants of P. *atrosepticum* strain SCRI1043, a time-dependent experiment with samplings at different time points was performed for selected 10 *P. atrosepticum* transposon mutants that showed greater fold change of GUS activity (PbaTn5-48, PbaTn5-B52, PbaTn5-43, PbaTn5-A27, PbaTn5-38, PbaTn5-B76 at 18 °C and PbaTn5-A2, PbaTn5-A33, PbaTn5-B25, PbaTn5-B42 at 28 °C) as described by Ullrich et al. [59]. The shift of bacterial cultures from non-inductive to inductive temperatures takes ca. 10 min, and this time was excluded from the total assay time. Of the 10 *P. atrosepticum* Tn5 mutants tested, all expressed a steady increase in GUS activity upon transfer from one temperature to another. Figure 5 illustrates the results for the selected *P. atrosepticum* transposon mutants with a similar level of GUS activity. Generally, the transposon mutants displayed a remarkable lag phase for GUS induction (2–7 h), that bacteria needed to adapt to the new temperature regime before GUS expression was initiated. The above is in line with results obtained by Ullrich et al. [59] from experiments with *P. syringae* pv. *glycinea*. 

## 3. Discussion

Several studies reported that the increasing average temperatures throughout the growing season due to climate change are recognized as one of the main reasons for a shift in the distribution of *Dickeya* spp. and *Pectobacterium* spp. on potato in Europe [23,27,30,35,66,67]. Although SRPs have been studied for decades, little is still known about temperature-responsive genes and thermoregulation of gene expression in *Dickeya* spp. and *Pectobacterium* spp. Our previous study has described the influence of temperature on gene expression in *D. solani* [61]. In the present study, we first attempted to establish a relation between temperature and regulation of gene expression, especially with regard to pathogenicity, in *P. atrosepticum*.

Out of the 5775 transposon mutants of *P. atrosepticum*, SCRI1043 examined, only 40 mutants (less than 1% of the total number of the obtained mutants) appear to have transposon insertions in temperature-dependent genes. The low number of the identified temperature-responsive loci in *P. atrosepticum* is consistent with our previous results obtained for *D. solani* [61] and the findings of similar studies on *Pseudomonas syringae* pv. *glycinea* and *Erwinia amylovora* [59,60]. 

In our previous investigation, the shift in growth temperature to a cool temperature of 18 °C altered gene expression levels, measured by GUS activity in relatively only a few mutants of *D. solani* strain IFB0099. Only nine transposon mutants out of 54 mutants of *D. solani* showed a higher GUS activity at 18 °C [61]. Contrary, in *P. atrosepticum* strain SCRI1043, the number of transposon mutants with higher expression of the genes, with the introduced GUS cassette, at 18 °C was 20, the same as the number of mutants with a higher GUS activity at 28 °C. It might be related to the fact that *P. atrosepticum* is a “cold-weather” pathogen and is thus well adapted to cool temperatures. *D. solani* grows in a wide range of temperatures [27], and the optimal temperature for its growth is relatively higher: 35 °C [26]. According to these data, it is reasonable that in the cells of *D. solani*, the expression of virulence factors is induced at higher temperatures than in *P. atrosepticum*. This finding is consistent with the previous studies that have examined the effect of temperature on plant cell wall degrading enzyme (PCWDE) in plant pathogenic bacteria. Smadja et al. [57] demonstrated that the pectate lyase activity of *P. atrosepticum* was maximal at 12 °C, and protease activity was induced at 17–24 °C. In the case of *D. solani*, Golanowska et al. [27] showed that this bacterium has the highest pectinolytic, cellulolytic and proteolytic activities at 28 °C rather than at lower temperatures. Also, in closely related *D. dadantii* strain 3937, the expression of *pel* genes encoding pectate lyases was maximal at 25 °C [52].

The possible functions for 29 temperature-regulated *P. atrosepticum* loci from this study were identified based on the comparison of their sequences with the sequences available in the GenBank database. The transposon was evenly inserted in different genes of the *P. atrosepticum* SCRI1043 genome, suggesting genome-wide insertion. Mutations targeted genes coding for proteins involved in fundamental bacterial metabolism, regulatory proteins, membrane proteins, and hypothetical proteins. However, the roles of most of them in adaptation to temperature fluctuations and infection of host plants are unclear.

The temperature-induced loci of *P. atrosepticum* identified in this study were compared to temperature-responsive loci of other plant pathogenic bacterial species, including *D. solani*, *P. syringae* pv. *glycinea*, and *E. amylovora* were identified using similar techniques [59,60,61] and *E. coli* [68]. Interestingly, several similar genetic loci were induced at the same temperature despite the different life strategies of these bacteria and their adaptation to other hosts.

In *P. atrosepticum*, the genes associated with transcription, carbohydrate transport and metabolism, cell wall and membrane biogenesis, as well as signal transduction were up-regulated at low temperatures. In *P. atrosepticum*, but not in *D. solani*, temperature-dependent *gusA* expression was observed in the mutants with a transposon insertion in genes involved in biosynthesis, transport, and catabolism of secondary metabolites. Transposon mutant, PbaTn5-A6, harbored a Tn5 insertion in a gene encoding DNA-binding protein H-NS and showed a higher GUS activity at 18 °C. It has previously been demonstrated that H-NS plays an essential role in the adaptation of *E. coli* to low temperatures [69,70]. Nasser et al. [71] indicated that H-NS plays a crucial role in the regulation of the pathogenicity of *D. dadantii*. They demonstrated that a *hns* mutant of *D. dadantii* displayed reduced growth rate, motility, and virulence on plants but increased exopolysaccharides (EPS) synthesis [71]. In this study, the maceration ability of the mutant PbaTn5-A6 on potato tubers was slightly decreased, and the biofilm formation ability was increased compared to the wild-type strain. No difference in growth rates was observed between the mutant PbaTn5-A6 and the wild-type strain *P. atrosepticum* SCRI1043.

The transposon mutant PbaTn5-4 with a transposon insertion in the gene encoding ClpB protein showed an increased GUS expression at 28 °C. Previously it has been indicated that *clpB* gene expression is induced in response to heat stress and is required for growth at high temperatures [72,73]. Therefore, this finding of the current study confirms that *P. atrosepticum* grows better at lower temperatures (<25 °C), and a temperature around 28 °C is sufficient to induce the synthesis of ClpB protein.

Although the majority of temperature-dependent loci identified in this study were not associated directly with the virulence of *P. atrosepticum*, it is still possible that other unidentified bacterial loci could be implicated in its virulence. Forty transposon mutants of *P. atrosepticum* SCRI1043 were tested to investigate whether mutations in the identified genetic loci affected their phenotypic characteristics associated with the ability to cause potato tuber maceration. The majority of mutations in temperature-responsive loci did not have any noticeable effect on the phenotypes of the *P. atrosepticum* Tn5 mutants. Only five thermoresponsive transposon mutants of *P. atrosepticum* SCRI1043 displayed discriminative phenotypes (e.g., low maceration ability, decreased biofilm formation, lack of PCWDE activities) from the phenotype of the wild-type *P. atrosepticum* strain SCRI1043:

Mutant PbaTn5-B76: had a mutation in the region, homologous to the *galU* gene encoding UTP-glucose-1-phosphate uridylyltransferase, which catalyzes the formation of UDP-glucose from glucose-1-phosphate and UTP [74]. In *E. coli*, UDP-glucose is an essential intermediate for growth on galactose and trehalose and is involved in the biosynthesis of carbohydrates [75]. Mutation in the *galU* gene reduced the virulence of many bacterial pathogens, for instance, *E. coli* [76], *P. aeruginosa* [77], *P. syringae* [78], *Vibrio cholerae* [79], *X. citri* subsp. *citri* [80], and the secretion of α-hemolysin and *D. dadantii* protease expression in *E. coli* [81]. These defects in virulence and secretion might be a consequence of the defect in lipopolysaccharide [81]. This study showed that the expression of the *galU* gene had a 1.6-fold increase at 18 °C compared to 28 °C. This finding is in line with the results of White-Ziegler et al. [68], which showed that a lower temperature (23 °C) increases the expression of the *galU* gene in *E. coli*. This study found that the PbaTn5-B76 mutant showed reduced pectate lyase, cellulase, and protease activity and exhibited reduced virulence on potato tubers. It can be speculated that the *galU* mutant of *P. atrosepticum* produces the truncated LPS, which enhances the surface hydrophobicity of these mutants, which may have resulted in increased autoaggregation and enhanced biofilm formation.

For the mutant PbaTn5-A43, the possible association of identified mutated genetic locus with their ability to macerate plant tissue is not so straightforward. In this mutant, the transposon insertion has been localized in gene encoding protein, lacking homologs in the other bacterial species, explaining its function. The mutant PbaTn5-A43 displayed enhanced virulence on potato tuber slices compared to the wild-type strain. We can only hypothesize that this hypothetical protein could be involved in the pathogenesis of *P. atrosepticum*, but further experimental analysis is required to establish its ecological relevance.

Mutant PbaTn5-43 carried transposon insertion in the gene related to the biosynthesis of LPS. It was shown that the *wba* gene was up-regulated at 18 °C. The WbaP (formerly RfbP) protein is a UDP-phosphate galactose phosphotransferase involved in the synthesis of the core oligosaccharide and O-antigen [82]. The *wba* mutant of *S. enterica* produced LPS lacking full-length O-antigen [83]. Also, the mutants of *Vibrio fischeri* that contained a mutation in the gene encoding a putative undecaprenyl-phosphate galactose phosphotransferase had an increased biofilm formation ability [84]. 

Mutant PbaTn5-B58: carried transposon insertion in the gene related to the biosynthesis of EPS. In this study, the *wza* gene was shown to be up-regulated at 28 °C. Many bacteria produce EPS and capsular polysaccharides (CPS) that play crucial roles during the infection process in human and animal pathogens [85]. In plant pathogenic bacteria, the production of EPS on plant surfaces or tissues allows bacterial colonization and biofilm formation [86]. Wza is an outer membrane lipoprotein of the outer membrane auxiliary (OMA) family of proteins, which is essential for the export and assembly of CPS and EPS in *E. coli* [85,87]. In this study, the *wza* mutant in PbaTn5-B58 exhibited an increased biofilm formation on polypropylene surfaces as well as displaying a decreased growth rate on M9 minimal medium supplemented with 0.4% glycerol. These results are consistent with those of Yi et al. [88], who showed that the *wza* deficient mutant of *Riemerella anatipestifer* grew slowly, had a significantly increased biofilm formation capacity, and exhibited enhanced autoaggregation compared to the wild-type strain. It is possible that the increased hydrophobicity of the *wza* mutant may affect the increased biofilm formation capacity [88]. On the contrary, a different study showed that transposon mutants of *Klebsiella pneumoniae* with insertions in *wza* locus were deficient in biofilm formation [89].

Mutant PbaTn5-B31: inactivation of the gene *rbsK* responsible for the ribokinase synthesis demonstrated a reduction in biofilm formation compared to the wild-type strain. Ribokinase (RbsK) catalyzes the conversion of ribose to ribose 5-phosphate [90,91]. The *rbsK* gene is part of the *rbs* operon, which also involves the high-affinity ribose transport system [91]. Following the present result, the previous study has demonstrated that the *rbsK* mutant of *E. coli* showed a two-fold decrease in the level of biofilm formation compared to the wild-type [92]. Also, the *rbsK* gene of *S. aureus* was found to be downregulated in a biofilm in comparison to the stationary phase of planktonic growth [93]. Furthermore, the observed phenotype of this mutant could result from polar effect on the downstream *rbsR* gene encoding the repressor of the ribose operon, which was confirmed for the *rbsK* mutant of *Serratia* sp. [94].

Of the 29 temperature-regulated *P. atrosepticum* loci in detail characterized in this study, 14 were predicted to be expressed as parts of the operons, whereas 15 others were expected to be transcribed as single genes. The knowledge of the molecular basis of *P. atrosepticum* temperature-regulated gene expression is scarce. As the genes arranged in operons are in the majority functionally related to each other and/or regulated in a sequential manner, it is interesting to see that in *P. atrosepticum* SCRI1043, at least some of them, as demonstrated in this study, are also differentially expressed according to the temperature. 

The obtained result indicated that several loci essential for the virulence of *P. atrosepticum* were identified. Further analysis of these genes will improve our knowledge about temperature-dependent mechanisms important for these plant-pathogenic bacteria during disease development. Future research could reveal the functions of these genes in the ecology and pathogenicity of this pathogen. However, it can be speculated that the temperature-induced loci identified in this study may also play a role in the ecological adaptation and fitness of *P. atrosepticum* and may also favor the survival of bacteria in unfavorable environmental conditions. It is important to note that many of the differentially regulated genes in response to the temperature code for hypothetical proteins that have an unknown function, and thereby these hypothetical proteins may possess novel physiological roles associated with thermoregulation.

## 4. Materials and Methods

### 4.1. Bacterial Strains and Media Used

*P. atrosepticum* SCRI1043 was grown at 28 °C with aeration (140 rpm) in tryptic soy broth (TSB; Oxoid, Basingstoke, UK) or in M9 minimal medium (MP Biomedicals, Thüringen, Germany) prepared as described by Czajkowski et al. [61] containing 2 mM magnesium sulfate (Sigma-Aldrich, Darmstadt, Germany), 0.1 mM calcium chloride (Sigma-Aldrich, Darmstadt, Germany), and 0.4% glucose (Sigma-Aldrich, Darmstadt, Germany) as a carbon source, pH 7. To solidify the media, 15 g L^−1^ agar (Oxoid, Basingstoke, UK) was added. *Escherichia coli* S17-1 λ-*pir* strain carrying plasmid pCAM140 with mini-Tn5 transposon [64] was cultured with aeration (120 rpm) in TSB or on tryptic soy agar (TSA; Oxoid, Basingstoke, UK) supplemented with ampicillin (Sigma-Aldrich, Darmstadt, Germany) to a final concentration of 100 μg mL^−1^ at 37 °C. When required, the bacterial media were supplemented with streptomycin (Sigma-Aldrich, Darmstadt, Germany) to a final concentration of 50 μg mL^−1^ and with X-Gluc (5-bromo-4-chloro-3-indolyl-β-D-glucuronic acid; GeneON, Ludwigshafen am Rhein, Germany) to a final concentration of 20 μg mL^−1^. For long-term usage, bacterial strains were stored in 40% (*v/v*) glycerol at −80 °C.

### 4.2. Transposon Mutagenesis of P. atrosepticum SCRI1043

Random transposon mutagenesis by mini-Tn5 transposon was carried out by conjugation of *P. atrosepticum* strain SCRI1043 with *E. coli* S17-1 λ-*pir* containing pCAM140 at 28 °C as described previously [61,95]. Suicide plasmid pCAM140 harbors a mini-Tn5 transposon that has a promotorless *β*-glucuronidase gene (*gusA*). This plasmid can be replicated in *E. coli* S17-1 λ-*pir* but not in *P. atrosepticum* cells [64]. The efficiency of Tn5 transfer was defined as the ratio of the number of obtained mutants to the total number of recipient *P. atrosepticum* cells after 6 h of mating with a 1:1 ratio of donor to recipient. The experiment was independently repeated three times, and the results were averaged.

### 4.3. Identification of P. atrosepticum Tn5 Mutants by PCR and Plating on Selective CVP Medium

PCR detection of *P. atrosepticum* transposon mutants was performed using the previously described colony PCR procedure [61] according to Frechon et al. [96] using primers Y45 (5′-TCACCGGACGCCGAACTGTGGCGT-3′, Genomed, Warsaw, Poland) and Y46 (5′-TCGCCAACGTTCAGCAGAACAAGT-3′, Genomed, Warsaw, Poland). These primers amplify a 439 bp fragment exclusively from strains of *P. atrosepticum*. The presence of transposon in *P. atrosepticum* SCRI1043 mutants was additionally confirmed by PCR using primers gusAf (5′-ACGTCCTGTAGAAACCCCAAC-3′, Genomed, Warsaw, Poland) and gusAr (5′-TTGTCCAGTTGCAACCACCT-3′, Genomed, Warsaw, Poland), which amplified a 679 bp fragment of the *gusA* gene located in the miniTn5 transposon. Amplified DNA fragments were detected by electrophoresis on a 1% 0.5× TBE agarose gel stained with 50 μg mL^−1^ GelRed (Biotium, Fremont, CA, USA). The ability of Tn5 mutants to form characteristic cavities (pits) specific for SRP on crystal violet pectate medium (CVP) was tested as described by Helias et al. [97].

### 4.4. Visual Estimation of β-glucuronidase Activity of P. atrosepticum Tn5 Mutants

The *β*-glucuronidase (GUS) activity of the Tn5 bacterial mutants was estimated visually by the development of blue color of bacterial colonies growing at 18 and 28 °C on M9 agar plates supplemented with streptomycin to a final concentration of 50 μg mL^−1^ and X-Gluc to a final concentration of 20 μg mL^−1^. Intensities of blue color formation were compared daily for a total time of 4 days. The experiment was independently repeated twice with the same setup. Mutants expressing the identical phenotypes at two selected temperatures (e.g., white at 18 °C and white at 28 °C or blue at 18 °C and blue at 28 °C) were removed from further analyses. 

### 4.5. Semi-Quantitative Assays to Assess the Rate of Gene Expression in P. atrosepticum Tn5 Mutants

The *β*-glucuronidase activity was quantified by a spectrophotometric assay with p-nitrophenol-*β*-D-glucuronide (Sigma-Aldrich, Darmstadt, Germany) as a substrate for *β*-glucuronidase with following fluorometric assay using 4-methylumbelliferyl-*β*-D-glucuronide (Merck, Warsaw, Poland) as a substrate for *β*-glucuronidase as previously described [61]. Total protein concentration was determined using the Bradford method [98] with a Pierce BCA Protein Assay kit (Thermo Scientific, Warsaw, Poland). The *β*-glucuronidase activity of the Tn5 mutants was measured as a pmol product (p-nitrophenol or 4-methyl umbelliferone) per min per µg total protein. Mutants showing statistically significant differences in *β*-glucuronidase activity at either temperature were selected and retested under the same conditions with four replicates per isolate and used in the follow-up studies.

### 4.6. Identification of Regions Flanking the Tn5 Transposon Insertion

Genomic DNA from *P. atrosepticum* transposon mutants was isolated according to procedures described by Sambrook et al. [99]. The flanking sequences of the insertion site were obtained by sequencing from the O-end and I-end of the miniTn5 with the primers OendB 5′-TTTCTACAGGACGTAACATAAGGG-3′ [100] and IendB 5′-GGGAATTCGGCCTAGGCGG-3′ [101]. DNA sequencing was performed at the Laboratory of DNA Sequencing and Oligonucleotide Synthesis at the Institute of Biochemistry and Biophysics of the Polish Academy of Science, Warsaw, Poland. Obtained sequences were compared with available sequences of bacterial genes deposited in GenBank using the BLASTN and BLASTX alignments (https://blast.ncbi.nlm.nih.gov/Blast.cgi, accessed: January–July 2018).

### 4.7. Phenotypic Characterization of P. atrosepticum Transposon Mutants

*P. atrosepticum* Tn5 transposon mutants that showed at least 1.5-fold-increased GUS activity in a temperature-dependent manner were characterized further for their ability to swim on motility agar [102], to produce pectate lyases [103], cellulases [104], proteases [105], ability to form a biofilm [106] and to cause rotting of potato tubers [107]. The phenotypic tests were done at 28 °C. 

### 4.8. Measurement of Bacterial Growth Rates

Growth rates of *P. atrosepticum* transposon mutants were measured using EnVision Multilabel Reader (Perkin Elmer, Baesweiler, Germany). Overnight bacterial cultures, grown at 28 °C, were diluted at 1:50 with fresh M9 medium supplemented with 0.4% glucose or 0.4% glycerol, and 0.5 mL of the diluted culture was added to the internal wells of 48-well microtiter plates (Becton Dickinson Labware, Temse, Belgium). Plates were sealed using optical clear sealing tape (Sarstedt, Warsaw, Poland) and incubated at 28 °C with shaking (orbital, 60 rpm). The growth was recorded spectrophotometrically at 600 nm wavelength every hour for the total incubation time of 16 h. The growth of each *P. atrosepticum* transposon mutant was analyzed in six replicates, and the results were averaged per strain. Each 48-well plate contained six negative (non-inoculated growth medium) and six positive (wild-type *P. atrosepticum* SCRI1043 culture) wells as controls. A Growthcurver package in R [108] was used to calculate growth rates and the generation (doubling) time. The experiment was independently repeated once with the same setup.

### 4.9. LPS Extraction and Analysis

Crude lipopolysaccharides (LPS) were extracted from an equivalent number of bacterial cells (5 McF), as described by Apicella et al. [109]. Samples were analyzed using NuPAGE^TM^ 4–12% Bis-Tris gels (1.0 mm, 12-well) and the corresponding NuPAGE MOPS SDS running buffer (20×) from Thermo Scientific, Warsaw, Poland. Silver staining was performed as described by Tsai and Frasch [110].

### 4.10. Morphological Characterization of the P. atrosepticum SCRI1043 Tn5 Mutants by Electron Microscopy (TEM)

Bacteria were grown overnight in TSB at 28 °C with shaking (200 rpm). TEM analysis was performed by the Laboratory of Electron Microscopy (Faculty of Biology, University of Gdansk, Poland). For the TEM analysis, bacteria were adsorbed onto carbon-coated grids (Sigma-Aldrich, Darmstadt, Germany), stained with 1.5% uranyl acetate, and directly examined with an electron microscope (Tecnai Spirit BioTWIN, FEI, New York, NY, USA) as described by Czajkowski et al. [111]. At least 10 photos were taken per strain to assess the morphology of Tn5 bacterial mutants.

### 4.11. Time-Dependent Induction of Gene Expression in Tn5 Mutants

Bacterial cultures of *P. atrosepticum* transposon mutants were cultivated in liquid in 30 mL M9 minimal medium supplemented with 0.4% glucose at the respective non-inductive temperature in the incubator with temperature control and shaking (140 rpm) until the cell density reached an OD600 of 1.0. Afterwards, the bacterial cultures were shifted to the growth temperature known to induce GUS activity in the respective mutant. Samples were taken at 1–2 h intervals for the total time of 9 h after the shift, and GUS activity was measured using the fluorometric GUS assay.

### 4.12. Statistical Analysis

Statistical analysis of data was performed using the R software [112]. At least two independent biological replicates were analyzed in each experiment. Error bars in the figures indicate standard deviation. Levene’s test [113] was applied for testing the equality of variances, and Shapiro–Wilk’s test [114] was implemented for evaluating the normality of the data. A Student’s *t*-test was performed to determine whether there was a significant difference in the different phenotypes between the wild-type and the transposon mutants. Significance was defined as a *p*-value lower than 0.05. The Wilcoxon–Mann–Whitney test [115] was used instead of a *t*-test when the data were not normally distributed.

## Figures and Tables

**Table 1:** Description of thermoresponsive loci of *P. atrosepticum* SCRI1043 transposon mutants.

Number	Mutant	% Identity ^a^; Protein, Accession Number; Gene ^b^; TU ^c^	Predicted Function	Fold Induction ^d^ of GUS Activity	Function Group (COGs) ^e^	Differential Phenotype
**Mutants with increased GUS activity at 18 °C**						
1	PbaTn5-38	99; CAG74583.1; *ECA_RS08290*; PTU	Transcriptional regulator	1.6	COG2336T	WT ^f^
2	PbaTn5-43	100; WbaP; CAG74330.1; *ECA_RS07075* (*rfbP*); MTU	UDP-phosphate galactose phosphotransferase	2.0	COG2148M	enhanced biofilm formation, reduced growth
3	PbaTn5-A6	95; H-NS; CAG75793.1; *ECA_RS14220*; MTU	DNA-binding protein H-NS	1.5	COG2916S ENOG501RF2B	WT
4	PbaTn5-A21	98; CAG73240.1; *ECA_RS01655*; PTU	Putative phosphoheptose isomerase	1.5	COG0279G	WT
5	PbaTn5-A27	100; CAG74551.1; *ECA_RS08155*; MPU	Hypothetical protein ECA1647	1.9	ENOG502FKDA	WT
6	PbaTn5-A29	100; CAG74078.1; *ECA_RS05790*; PTU	Putative 50S ribosomal protein L31	1.6	COG0254J	WT
7	PbaTn5-B9	90; CAG73972.1; *ECA_RS05265*; PTU	Putative integrase	2.1	COG4688S	WT
8	PbaTn5-B16	100; CAG75691.1; *ECA_RS13700*; MTU	Putative glutatione S-transferase	1.5	COG0625O	WT
9	PbaTn5-B31	100; CAG72938.1; *ECA_RS00065*; PTU	Ribokinase	2.6	COG0524G	reduced biofilm formation
10	PbaTn5-B36	100; CAG77284.1; *ECA_RS21710*; PTU	IIABC component of phosphoenolpyruvate-dependent sugar phosphotransferase (PTS) system	1.5	COG1263G	WT
11	PbaTn5-B52	100; AIK15419.1; *ECA_RS18040*; MTU	Transcriptional regulator, XRE family	2.2	ENOG501MXWB	WT
12	PbaTn5-B63	100; CAG77013.1; *ECA_RS20345*; MTU	Putative IucA/IucC family siderophore biosynthesis protein	2.0	COG4264P	WT
13	PbaTn5-B76	96; GalU; CAG75232.1; *ECA_RS11440* (*galU*); MTU	UTP-glucose-1-phosphate uridylyltransferase	1.6	COG1210M	reduced swimming motility, reduced exoenzyme production, reduced growth, enhance biofilm formation, reduced ability to macerate potato tissue, altered LPS synthesis, altered lactose fermentation
14	PbaTn5-B78	100; MucB, RseB; CAG76180.1; *ECA_RS16150*; MTU	Sigma-E factor regulatory protein	1.8	COG3026T	WT
**Mutants with increased GUS activity at 28 °C**						
15	PbaTn5-1	98; CAG73522.1; *ECA_RS03020*; PTU	Cfa-β-ketoacyl synthase	2.2	COG0304IQ	WT
16	PbaTn5-4	100; ClpB; CAG76243.1; *ECA_RS16515*; MTU	Chaperone protein ClpB	2.8	COG0542O	WT
17	PbaTn5-8	100; CAG76476.1; *ECA_RS17680*; MTU	d-galactarate dehydratase	2.9	COG2721G	WT
18	PbaTn5-11	85; CAG74973.1; *ECA_RS10210*; PTU	Putative cytochrome P450	3.8	COG2124Q	WT
19	PbaTn5-16	100; CAG76859.1; *ECA_RS19560*; MTU	Putative exported protein	2.0	ENOG502C5YQ	WT
20	PbaTn5-26	91; CAG75749.1; *ECA_RS13980*; MTU	Metallo-β-lactamase	2.8	COG0491GM	WT
21	PbaTn5-A2	93; CAG73973.1; *ECA_RS05270*; PTU	Conserved hypothetical protein	5.0	ENOG502E3II	WT
22	PbaTn5-A33	89; CAG75864.1; *ECA_RS14560*; MTU	Amidohydrolase; putative peptidase	4.7	COG1473S	WT
23	PbaTn5-A43	100; CAG76360.1; *ECA_RS17050*; PTU	Putative exported protein	2.3	ENOG502ASC5	enhanced ability to macerate potato tissue
24	PbaTn5-B25	77; HybO; CAG74135.1; *ECA_RS06065*; PTU	Hydrogenase-2 small subunit	2.7	COG1740C	WT
25	PbaTn5-B30	100; CAG73872.1; *ECA_RS04750*; MTU	AAA family ATPase	2.2	COG0419L	WT
26	PbaTn5-B42	99; WP_011091854.1; *ECA_RS01110*; MTU	Elongation factor Tu	2.3	COG0050J	WT
27	PbaTn5-B53	99; CAG76514.1; *ECA_RS17865*; PTU	RNA ligase RtcB family protein	1.6	COG1690J	WT
28	PbaTn5-B58	98; Wza; CAG74327.1; *ECA_RS07060*; PTU	Putative polysaccharide export protein	1.7	COG1596M	enhanced biofilm formation, reduced growth
29	PbaTn5-B80	100; CAG73490.1; *ECA_RS02860*; PTU	Putative membrane protein	1.8	ENOG5028UFW	WT

^a^ Amino acid sequence identity. ^b^ Gene name according to the gene nomenclature for *P. atrosepticum* SCRI1043. ^c^ TU–transcription unit, identified using the BioCyc database (biocyc.org); PTU–polycistronic transcription unit, MTU–monocistronic transcription unit. ^d^ Fold induction at 18 °C was calculated by dividing the GUS activity at 18 °C by the GUS activity at 28 °C, and fold induction at 28 °C was calculated by dividing the GUS activity at 28 °C by the GUS activity at 18 °C, as measured by the fluorometric assay. ^e^ Functional characterization of the proteins was predicted by the software EggNOG5.0.0. ^f^ Phenotype not significantly different than in wild-type *P. atrosepticum* strain SCRI1043.

## Data Availability

Data are contained within the article and supplementary material.

## Supplementary Materials

The following are available online at https://www.mdpi.com/article/10.3390/ijms22094839/s1.

## References

[1] FAOSTAT. http://www.fao.org/faostat/en/#data/QC.

[2] Perombelon M.C.M. (2002). Potato diseases caused by soft rot *Erwinias*: An overview of pathogenesis. Plant Pathol..

[3] Czajkowski R., Grabe G.J., van der Wolf J.M. (2009). Distribution of *Dickeya* spp. and *Pectobacterium carotovorum* subsp. *carotovorum* in naturally infected seed potatoes. Eur. J. Plant Pathol..

[4] Toth I.K., van der Wolf J.M., Saddler G., Lojkowska E., Helias V., Pirhonen M., Tsror (Lahkim) L., Elphinstone J.G. (2011). *Dickeya* species: An emerging problem for potato production in Europe. Plant Pathol..

[5] Gardan L., Gouy C., Christen R., Samson R. (2003). Elevation of three subspecies of *Pectobacterium carotovorum* to species level: *Pectobacterium atrosepticum* sp. nov., *Pectobacterium betavasculorum* sp. nov. and *Pectobacterium wasabiae* sp. nov. Int. J. Syst. Evol. Microbiol..

[6] Khayi S., Cigna J., Chong T.M., Quetu-Laurent A., Chan K.-G., Helias V., Faure D. (2016). Transfer of the potato plant isolates of *Pectobacterium wasabiae* to *Pectobacterium parmentieri* sp. nov. Int. J. Syst. Evol. Microbiol..

[7] Dees M.W., Lysøe E., Rossmann S., Perminow J., Brurberg M.B. (2017). *Pectobacterium polaris* sp. nov., isolated from potato (*Solanum tuberosum*). Int. J. Syst. Evol. Microbiol..

[8] Sarfraz S., Riaz K., Oulghazi S., Cigna J., Sahi S.T., Khan S.H., Faure D. (2018). *Pectobacterium punjabense* sp. nov., isolated from blackleg symptoms of potato plants in Pakistan. Int. J. Syst. Evol. Microbiol..

[9] Pedron J., Bertrand C., Taghouti G., Portier P., Barny M.A. (2019). *Pectobacterium aquaticum* sp. nov., isolated from waterways. Int. J. Syst. Evol. Microbiol..

[10] Portier P., Pédron J., Taghouti G., Fischer-Le Saux M., Caullireau E., Bertrand C., Laurent A., Chawki K., Oulgazi S., Moumni M. (2019). Elevation of *Pectobacterium carotovorum* subsp. *odoriferum* to species level as *Pectobacterium odoriferum* sp. nov., proposal of *Pectobacterium brasiliense* sp. nov. and *Pectobacterium actinidiae* sp. nov., emended description of *Pectobacterium carotovorum* and description of *Pectobacterium versatile* sp. nov., isolated from streams and symptoms on diverse plants. Int. J. Syst. Evol. Microbiol..

[11] Waleron M., Misztak A., Waleron M., Franczuk M., Wielgomas B., Waleron K. (2018). Transfer of *Pectobacterium carotovorum* subsp. *carotovorum* strains isolated from potatoes grown at high altitudes to *Pectobacterium peruviense* sp. nov. Syst. Appl. Microbiol..

[12] Waleron M., Misztak A., Waleron M., Jonca J., Furmaniak M., Waleron K. (2019). *Pectobacterium polonicum* sp. nov. isolated from vegetable fields. Int. J. Syst. Evol. Microbiol..

[13] Pasanen M., Waleron M., Schott T., Cleenwerck I., Misztak A., Waleron K., Pritchard L., Bakr R., Degefu Y., van der Wolf J. (2020). *Pectobacterium parvum* sp. nov., having a *Salmonella* SPI-1-like type III secretion system and low virulence. Int. J. Syst. Evol. Microbiol..

[14] Samson R., Legendre J.B., Christen R., Fischer-Le Saux M., Achouak W., Gardan L. (2005). Transfer of *Pectobacterium chrysanthemi* (Burkholder et al. 1953) Brenner et al. 1973 and *Brenneria paradisiaca* to the genus *Dickeya* gen. nov. as *Dickeya chrysanthemi* comb. nov. and *Dickeya paradisiaca* comb. nov. and delineation of four novel species, *Dickeya dadantii* sp. nov., *Dickeya dianthicola* sp. nov, *Dickeya dieffenbachiae* sp. nov. and *Dickeya zeae* sp. nov. Int. J. Syst. Evol. Microbiol..

[15] Parkinson N., DeVos P., Pirhonen M., Elphinstone J. (2014). *Dickeya aquatica* sp. nov., isolated from waterways. Int. J. Syst. Evol. Microbiol..

[16] van der Wolf J.M., Nijhuis E.H., Kowalewska M.J., Saddler G.S., Parkinson N., Elphinstone J.G., Pritchard L., Toth I.K., Lojkowska E., Potrykus M. (2014). *Dickeya solani* sp. nov., a pectinolytic plant-pathogenic bacterium isolated from potato (*Solanum tuberosum*). Int. J. Syst. Evol. Microbiol..

[17] Toth I.K., Barny M., Czajkowski R., Elphinstone J.G., Li X., Pédron J., Pirhonen M., Van Gijsegem F., Van Gijsegem F., van der Wolf J.M., Toth I.K. (2021). Pectobacterium and *Dickeya*: Taxonomy and evolution. Plant Diseases Caused by Dickeya and Pectobacterium Species.

[18] Mansfield J., Genin S., Magori S., Citovsky V., Sriariyanum M., Ronald P., Dow M., Verdier V., Beer S.V., Machado M.A. (2012). Top 10 Plant Pathogenic Bacteria in Molecular Plant Pathology. Mol. Plant Pathol..

[19] Perombelon M.C.M., Kelman A. (1980). Ecology of the soft rot *Erwinias*. Annu. Rev. Phytopathol..

[20] Cother E.J., Gilbert R.L. (1990). Presence of *Erwinia chrysanthemi* in two major river systems and their alpine sources in Australia. J. Appl. Bacteriol..

[21] Laurila J., Hannukkala A., Nykyri J., Pasanen M., Hélias V., Garlant L., Pirhonen M. (2010). Symptoms and yield reduction caused by *Dickeya* spp. strains isolated from potato and river water in Finland. Eur. J. Plant Pathol..

[22] Tsror (Lahkim) L., Lebiush S., Erlich O., Ben-Daniel B., van der Wolf J. (2010). First report of latent infection of *Cyperus rotundus* cused by a biovar 3 *Dickeya* sp. (syn. *Erwinia chrysanthemi*) in Israel. New Dis. Rep..

[23] Potrykus M., Golanowska M., Sledz W., Zoledowska S., Motyka A., Kolodziejska A., Butrymowicz J., Lojkowska E. (2016). Biodiversity of *Dickeya* spp. isolated from potato plants and water sources in temperate climate. Plant Dis..

[24] Fikowicz-Krosko J., Wszalek-Rozek K., Smolarska A., Czajkowski R. (2017). First report on isolation of soft rot *Pectobacterium carotovorum* subsp. *carotovorum* from symptomless bittersweet nightshade occurring in rural area in Poland. J. Plant Pathol..

[25] Pulatov B., Linderson M.L., Hall K., Jönsson A.M. (2015). Modeling climate change impact on potato crop phenology, and risk of frost damage and heat stress in northern Europe. Agric. For. Meteorol..

[26] du Raan S., Coutinho T.A., van der Waals J.E. (2016). Cardinal temperature differences, determined *in vitro*, between closely related species and subspecies of pectinolytic bacteria responsible for blackleg and soft rot on potatoes. Eur. J. Plant Pathol..

[27] Golanowska M., Kielar J., Lojkowska E. (2017). The effect of temperature on phenotypic features and the maceration ability of *Dickeya solani* strains isolated in Finland, Israel and Poland. Eur. J. Plant Pathol..

[28] Janse J.D., Ruissen M.A. (1988). Characterization and classification of *Erwinia chrysanthemi* strains from several osts in the Netherlands. Phytopathology.

[29] Cazelles O., Schwärzel R. (1992). Survey of bacterial diseases caused by *Erwinia* in seed potato fields in western Switzerland. Rev. Suisse d’Agric..

[30] Degefu Y., Potrykus M., Golanowska M., Virtanen E., Lojkowska E. (2013). A new clade of *Dickeya* spp. plays a major role in potato blackleg outbreaks in north Finland. Ann. Appl. Biol..

[31] Motyka A., Zoledowska S., Sledz W., Lojkowska E. (2017). Molecular methods as tools to control plant diseases caused by *Dickeya* and *Pectobacterium* spp: A minireview. New Biotechnol..

[32] Tsror (Lahkim) L., Erlich O., Lebiush S., van der Wolf J., Czajkowski R., Mozes G., Sikharulidze Z., Ben-Daniel B. (2011). First report of potato blackleg caused by a biovar 3 *Dickeya* sp. in Georgia. New Dis. Rep..

[33] Ignatov A.N., Karlov A.N., Dzhalilov F.S. (2014). Spreading of the blackleg of potatoes in Russia caused by bacteria of *Dickeya* genus. Zaschita Karantin Rastenij.

[34] de Werra P., Bussereau F., Kellenberger I., Dupuis B., Schaerer S., Keiser A. (2015). Potato: The *Pectobacterium* empire strikes back. Agrar. Schweiz.

[35] van der Wolf J.M., de Haan E.G., Kastelein P., Krijger M., de Haas B.H., Velvis H., Mendes O., Kooman-Gersmann M., van der Zouwen P.S. (2017). Virulence of *Pectobacterium carotovorum* subsp. *brasiliense* on potato compared with that of other *Pectobacterium* and *Dickeya* species under climatic conditions prevailing in the Netherlands. Plant Pathol..

[36] Zoledowska S., Motyka A., Zukowska D., Sledz W., Lojkowska E. (2018). Population structure and biodiversity of *Pectobacterium parmentieri* isolated from potato fields in temperate climate. Plant Dis..

[37] Oulghazi S., Sarfraz S., Zaczek-Moczydłowska M.A., Khayi S., Ed-Dra A., Lekbach Y., Campbell K., Moleleki L.N., O’hanlon R., Faure D. (2021). *Pectobacterium brasiliense*: Genomics, host range and disease management. Microorganisms.

[38] Śledź W., Jafra S., Waleron M., Łojkowska E. (2000). Genetic diversity of *Erwinia carotovora* strains isolated from infected plants grown in Poland. Bull. OEPP/EPPO Bull..

[39] Dees M.W., Lebecka R., Perminow J.I.S., Czajkowski R., Grupa A., Motyka A., Zoledowska S., Sliwka J., Lojkowska E., Brurberg M.B. (2017). Characterization of *Dickeya* and *Pectobacterium* strains obtained from diseased potato plants in different climatic conditions of Norway and Poland. Eur. J. Plant Pathol..

[40] Skelsey P., Humphris S.N., Campbell E.J., Toth I.K. (2018). Threat of establishment of non-indigenous potato blackleg and tuber soft rot pathogens in Great Britain under climate change. PLoS ONE.

[41] van der Wolf J.M., Acuña I., De Boer S.H., Brurberg M.B., Cahill G., Charkowski A.O., Coutinho T., Davey T., Dees M.W., Degefu Y., Van Gijsegem F., van der Wolf J.M., Toth I.K. (2021). Diseases caused by *Pectobacterium* and *Dickeya* species around the world. Plant Diseases Caused by Dickeya and Pectobacterium Species.

[42] Zaczek-Moczydłowska M.A., Fleming C.C., Young G.K., Campbell K., O’Hanlon R. (2019). *Pectobacterium* and *Dickeya* species detected in vegetables in Northern Ireland. Eur. J. Plant Pathol..

[43] Perombelon M.C.M. (1992). Potato blackleg: Epidemiology, host-pathogen interaction and control. Neth. J. Plant Pathol..

[44] Stommel J.R., Goth R.W., Haynes K.G., Kim S.H. (1996). Pepper (*Capsicum annum*) soft rot caused by *Erwinia carotovora* subsp. atroseptica. Plant Dis..

[45] Baştaş K.K., Hekimhan H., Maden S., Tör M. (2009). First report of bacterial stalk and head rot disease caused by *Pectobacterium atrosepticum* on sunflower in Turkey. Plant Dis..

[46] Ma B., Hibbing M.E., Kim H., Reedy R.M., Yedidia I., Breuer J., Breuer J., Glasner J.D., Perna N.T., Kelman A. (2007). Host range and molecular phylogenies of the soft rot *Enterobacterial* genera *Pectobacterium* and *Dickeya*. Phytopathology.

[47] Glasner J.D., Kim H., Jahn C.E., Ma B., Biehl B.S., Rissman A.I., Mole B., Yi X., Yang C., Dangl J.L. (2008). Niche-specificity and the variable fraction of the *Pectobacterium* pan-genome. MPMI.

[48] De Boer S.H., Li X., Ward L.J. (2012). *Pectobacterium* spp. associated with bacterial stem rot syndrome of potato in Canada. Phytopathology.

[49] Molina J.J., Harrison M.D. (1980). The role of *Erwinia carotovora* in the epidemiology of potato blackleg. II. The effect of soil temperature on disease severity. Am. Potato J..

[50] Ali H.F., Ahmad M., Junaid M., Bibi A., Ali A., Sharif M., Ali B., Nawab K., Sadozai A. (2012). Inoculum sources, disease incidence and severity of bacterial blackleg and soft rot of potato. Pak. J. Bot..

[51] Czajkowski R., De Boer W.J., Van der Zouwen P.S., Kastelein P., Jafra S., De Haan E.G., van den Bovenkamp G.W., van der Wolf J.M. (2013). Virulence of “*Dickeya solani*” and *Dickeya dianthicola* biovar-1 and -7 strains on potato (*Solanum tuberosum*). Plant Pathol..

[52] Hugouvieux-Cotte-Pattat N., Dominguez H., Robert-Baudouy J. (1992). Environmental conditions affect transcription of the pectinase genes of *Erwinia chrysanthemi* 3937. J. Bacteriol..

[53] Wei Z., Sneath B.J., Beer S.V. (1992). Expression of *Erwinia amylovora hrp* genes in response to environmental stimuli. J. Bacteriol..

[54] Ullrich M., Pen A., Bailey A., Bender C.L. (1995). A modified two-component regulatory system is involved in temperature-dependent biosynthesis of the *Pseudomonas syringae* phytotoxin coronatine. J. Bacteriol..

[55] van Dijk K., Fouts D.E., Rehm A.H., Hill A.R., Collmer A., Alfano J.R. (1999). The Avr (effector) proteins HrmA (HopPsyA) and AvrPto are secreted in culture from *Pseudomonas syringae* pathovars *via* the Hrp (Type III) protein secretion system in a temperature- and pH-sensitive manner. J. Bacteriol..

[56] Smirnova A., Li H., Weingart H., Aufhammer S., Burse A., Finis K., Schenk A., Ullrich M.S. (2001). Thermoregulated expression of virulence factors in plant-associated bacteria. Arch. Microbiol..

[57] Smadja B., Latour X., Trigui S., Burini J.F., Chevalier S., Orange N. (2004). Thermodependence of growth and enzymatic activities implicated in pathogenicity of two *Erwinia carotovora* subspecies (*Pectobacterium* spp.). Can. J. Microbiol..

[58] Lanham P.G., Mcllravey K.I., Perombelon M.C.M. (1991). Production of cell wall dissolving enzymes by *Erwinia carotovora* subsp. *atroseptica in vitro* at 27 °C and 30.5 °C. J. Appl. Bacteriol..

[59] Ullrich M.S., Schergaut M., Boch J., Ullrich B. (2000). Temperature-responsive genetic loci in the plant pathogen *Pseudomonas syringae* pv. *glycinea*. Microbiology.

[60] Goyer C., Ullrich M.S. (2006). Identification of low-temperature-regulated genes in the fire blight pathogen *Erwinia amylovora*. Can. J. Microbiol..

[61] Czajkowski R., Kaczyńska N., Jafra S., Narajczyk M., Lojkowska E. (2017). Temperature-responsive genetic loci in pectinolytic plant pathogenic *Dickeya solani*. Plant Pathol..

[62] Hinton J.C.D., Sidebotham J.M., Hyman L.J., Prombdon M.C.M., Salmond G.P.C. (1989). Isolation and characterisation of transposon-induced mutants of *Erwinia carotovora* subsp. *atroseptica* exhibiting reduced virulence. Mol. Gen. Genet..

[63] Bell K.S., Sebaihia M., Pritchard L., Holden M.T.G., Hyman L.J., Holeva M.C., Thomson N.R., Bentley S.D., Churcher L.J.C., Mungall K. (2004). Genome sequence of the enterobacterial phytopathogen *Erwinia carotovora* subsp. *atroseptica* and characterization of virulence factors. Proc. Natl. Acad. Sci. USA.

[64] Wilson K.J., Sessitsch A., Corbo J.C., Giller K.E., Akkermans D.L., Jefferson R. (1995). Beta-glucuronidase (Gus) transposons for ecological and genetic-studies of *Rhizobia* and other gram-negative bacteria. Microbiology.

[65] Huerta-Cepas J., Szklarczyk D., Heller D., Hernández-Plaza A., Forslund S.K., Cook H., Mende D.R., Letunic I., Rattei T., Jensen L.J. (2019). EggNOG 5.0: A hierarchical, functionally and phylogenetically annotated orthology resource based on 5090 organisms and 2502 viruses. Nucleic Acids Res..

[66] Haverkort A.J., Verhagen A. (2008). Climate change and its repercussions for the potato supply chain. Potato Res..

[67] Schaap B.F., Blom-Zandstra M., Hermans C.M.L., Meerburg B.G., Verhagen J. (2011). Impact changes of climatic extremes on arable farming in the north of the Netherlands. Reg. Environ. Chang..

[68] White-Ziegler C.A., Um S., Perez N.M., Berns A.L., Malhowski A.J., Young S. (2008). Low temperature (23 °C) increases expression of biofilm-, cold-shock- and RpoS-dependent genes in *Escherichia coli* K-12. Microbiology.

[69] Dersch P., Kneip S., Bremer E. (1994). The nucleoid-associated DNA-binding protein H-NS is required for the efficient adaptation of *Escherichia coli* K-12 to a cold environment. Mol. Gen. Genet..

[70] White-Ziegler C.A., Davis T.R. (2009). Genome-wide identification of H-NS-controlled, temperature-regulated genes in *Escherichia coli* K-12. J. Bacteriol..

[71] Nasser W., Faelen M., Hugouvieux-Cotte-Pattat N., Reverchon S. (2001). Role of the nucleoid-associated protein H-NS in the synthesis of virulence factors in the phytopathogenic bacterium *Erwinia chrysanthemi*. MPMI.

[72] Schirmer E.C., Glover J.R., Singer M.A., Lindquist S. (1996). HSP100/Clp proteins: A common mechanism explains diverse functions. Trends Biochem. Sci..

[73] Chan K.-G., Priya K., Chang C., Yamin A., Rahman A., Tee K.K., Yin W.-F. (2016). Transcriptome analysis of *Pseudomonas aeruginosa* PAO1 grown at both body and elevated temperatures. PeerJ.

[74] Thoden J.B., Holden H.M. (2007). The molecular architecture of glucose-1-phosphate uridylyltransferase. Protein Sci..

[75] Weissborn A.C., Liu Q., Rumley M.K., Kennedy E.P. (1994). UTP: α-D-glucose-1-phosphate uridylyltransferase of *Escherichia coli*: Isolation and DNA sequence of the *galU* Gene and purification of the enzyme. J. Bacteriol..

[76] Ho T.D., Waldor M.K. (2007). Enterohemorrhagic *Escherichia coli* O157:H7 *gal* mutants are sensitive to bacteriophage P1 and defective in intestinal colonization. Infect. Immun..

[77] Priebe G.P., Dean C.R., Zaidi T., Meluleni G.J., Coleman F.T., Coutinho Y.S., Noto M.J., Urban T.A., Pier G.B., Goldberg J.B. (2004). The *galU* gene of *Pseudomonas aeruginosa* is required for corneal infection and efficient systemic spread following pneumonia but not for infection confined to the lung. Infect. Immun..

[78] Deng W.-L., Lin Y.-C., Lin R.-H., Wei C.-F., Huang Y.-C., Peng H.-L., Huang H.-C. (2010). Effects of *galU* mutation on *Pseudomonas syringae*—plant interactions. MPMI.

[79] Nesper J., Lauriano C.M., Klose K.E., Kapfhammer D., Kraiß A., Reidl J. (2001). Characterization of *Vibrio cholerae* O1 El Tor *galU* and *galE* mutants: Influence on lipopolysaccharide structure, colonization, and biofilm formation. Infect. Immun..

[80] Guo Y., Sagaram U.S., Kim J., Wang N. (2010). Requirement of the *galU* gene for polysaccharide production by and pathogenicity and growth *in planta* of *Xanthomonas citri* subsp. *citri*. Appl. Environ. Microbiol..

[81] Wandersman C., Letoffe S. (1993). Involvement of lipopolysaccharide in the secretion of *Escherichia coli* α-haemolysin and *Erwinia chrysanthemi* proteases. Mol. Microbiol..

[82] Wang L.E.I., Reeves P.R. (1994). Involvement of the galactosyl-1-phosphate transferase encoded by the *Salmonella enterica rfbP* gene in O-antigen subunit processing. J. Bacteriol..

[83] Kong Q., Yang J., Liu Q., Alamuri P., Roland K.L., Curtiss R. (2011). Effect of deletion of genes involved in lipopolysaccharide core and O-antigen synthesis on virulence and immunogenicity of *Salmonella enterica* serovar typhimurium. Infect. Immun..

[84] Shibata S., Yip E.S., Quirke K.P., Ondrey J.M., Visick K.L. (2012). Roles of the structural symbiosis polysaccharide (*syp*) genes in host colonization, biofilm formation, and polysaccharide biosynthesis in *Vibrio fischeri*. J. Bacteriol..

[85] Cuthbertson L., Mainprize I.L., Naismith J.H., Whitfield C. (2009). Pivotal roles of the outer membrane polysaccharide export and polysaccharide copolymerase protein families in export of extracellular polysaccharides in gram-negative bacteria. Microbiol. Mol. Biol. Rev..

[86] Bogino P.C., Oliva M., de las M., Sorroche F.G., Giordano W. (2013). The role of bacterial biofilms and surface components in plant-bacterial associations. Int. J. Mol. Sci..

[87] Dong C., Beis K., Nesper J., Brunkan-LaMontagne A.L., Clarke B.R., Whitfield C., Naismith J.H. (2006). Wza the translocon for *E. coli* capsular polysaccharides defines a new class of membrane protein. Nature.

[88] Yi H., Yuan B., Liu J., Zhu D., Wu Y., Wang M., Jia R., Sun K., Yang Q., Chen S. (2017). Identification of a *wza*-like gene involved in capsule biosynthesis, pathogenicity and biofilm formation in *Riemerella anatipestifer*. Microb. Pathog..

[89] Wu M.-C., Lin T.-L., Hsieh P.-F., Yang H.-C., Wang J.-T. (2011). Isolation of genes involved in biofilm formation of a *Klebsiella pneumoniae* strain causing pyogenic liver abscess. PLoS ONE.

[90] Iida A., Harayama S., Iino T., Hazelbauer G.L. (1984). Molecular cloning and characterization of genes required for ribose transport and utilization in *Escherichia coli* K-12. J. Bacteriol..

[91] Hope J.N., Bell W., Hermodson M.A., Groarkeg J.M. (1986). Ribokinase from *Escherichia coli* K12. J. Biol. Chem..

[92] Romeo T., Wang X., Desplas R.L. (2005). Novel Genes Involved in the *Escherichia coli* Biofilm Formation and Uses Thereof. US Patent Appl. Publ..

[93] Beenken K.E., Dunman P.M., Mcaleese F., Macapagal D., Murphy E., Projan S.J., Blevins J.S., Smeltzer M.S. (2004). Global gene expression in *Staphylococcus aureus* biofilms. J. Bacteriol..

[94] Lee C.M., Monson R.E., Adams R.M., Salmond G.P.C. (2017). The LacI-family transcription factor, RbsR, is a pleiotropic regulator of motility, virulence, siderophore and antibiotic production, gas vesicle morphogenesis and flotation *in Serratia*. Front. Microbiol..

[95] Czajkowski R., Krzyzanowska D., Karczewska J., Atkinson S., Przysowa J., Lojkowska E., Williams P., Jafra S. (2011). Inactivation of AHLs by *Ochrobactrum* sp. A44 depends on the activity of a novel class of AHL acylase. Environ. Microbiol. Rep..

[96] Frechon D., Exbrayat P., Helias V., Hyman L.J., Jouan B., Llop P., Lopez M.M., Payet N., Perombelon M.C.M., Toth I.K. (1998). Evaluation of a PCR kit for the detection of *Erwinia carotovora* subsp. *atroseptica* on potato tubers. Potato Res..

[97] Hélias V., Hamon P., Huchet E., Wolf J.V.D., Andrivon D. (2012). Two new effective semiselective crystal violet pectate media for isolation of *Pectobacterium* and *Dickeya*. Plant Pathol..

[98] Bradford M.M. (1976). A rapid and sensitive method for the quantitation of microgram quantities of protein utilizing the principle of protein-dye binding. Anal. Biochem..

[99] Sambrook J., Fritsch E.F., Maniatis T. (1989). Molecular Cloning: A Laboratory Manual.

[100] Bittinger M.A., Handelsman J. (2000). Identification of genes in the *rosR* regulon of *Rhizobium etli*. J. Bacteriol..

[101] Yap M.-N., Yang C.-H., Charkowski A.O. (2008). The response regulator HrpY of *Dickeya dadantii* 3937 regulates virulence genes not linked to the *hrp* cluster. Mol. Plant. Microbe. Interact..

[102] Jahn C.E., Willis D.K., Charkowski A.O. (2008). The flagellar sigma factor FliA is required for *Dickeya dadantii* virulence. MPMI.

[103] Reverchon S., Van Gijsegem F., Rouve M., Kotoujansky A., Robert-Baudouy J. (1986). Organization of a pectate lyase gene family in *Erwinia chrysanthemi*. Gene.

[104] Py B., Bortoli-German I., Haiech J., Chippaux M., Barras F. (1991). Cellulase EGZ of *Erwinia chrysanthemi*: Structural organization and importance of His98 and Glul33 residues for catalysis. Protein Eng..

[105] Wandersman C., Andro T., Bertheau Y. (1986). Extracellular protease in *Erwinia chrysanthemi*. J. Gen. Microbiol..

[106] Nykyri J., Mattinen L., Niemi O., Adhikari S., Kõiv V., Somervuo P., Fang X., Auvinen P., Mäe A., Palva E.T. (2013). Role and regulation of the Flp/Tad pilus in the virulence of *Pectobacterium atrosepticum* SCRI1043 and *Pectobacterium wasabiae* SCC3193. PLoS ONE.

[107] Czajkowski R., de Boer W.J., Velvis H., van der Wolf J.M. (2010). Systemic colonization of potato plants by a soilborne, green fluorescent protein-tagged strain of *Dickeya* sp. biovar 3. Phytopathology.

[108] Sprouffske K., Wagner A. (2016). Growthcurver: An R package for obtaining interpretable metrics from microbial growth curves. BMC Bioinform..

[109] Apicella M.A., Griffiss J.M., Schneider H. (1994). Isolation and characterization of lipopolysaccharides, lipooligosaccharides, and lipid A. Methods Enzymol..

[110] Tsai C.M., Frasch C.E. (1982). A sensitive silver stain for detecting lipopolysaccharides in polyacrylamide gels. Anal. Biochem..

[111] Czajkowski R., van der Wolf J.M., Krolicka A., Ozymko Z., Narajczyk M., Kaczynska N., Lojkowska E. (2014). Salicylic acid can reduce infection symptoms caused by *Dickeya solani* in tissue culture grown potato (*Solanum tuberosum* L.) Plants. Eur. J. Plant Pathol..

[112] R Core Team (2016). R: A Language and Environment for Statistical Computing.

[113] Levene H. (1960). Robust tests for equality of variances. Contrib. Probab. Stat. Essays Honor Harold Hotell..

[114] Shapiro S.S., Wilk M.B. (1965). An analysis of variance test for normality (complete samples). Biometrika.

[115] Mann H.B., Whitney D.R. (1947). On a test of whether one or two random variables is stochastically larger than the other. Ann. Math. Stat..

