# Supplementary material for: Genome-Wide Analyses of the Temperature-Responsive Genetic Loci of the Pectinolytic Plant Pathogenic Pectobacterium atrosepticum
Source: Int J Mol Sci. 2021 May 3;22(9):4839. doi: 10.3390/ijms22094839 (PMC8125463; doi:10.3390/ijms22094839)
Supplement: Supplementary file 1 [file ijms-22-04839-s001.zip › ijms-1187187-supplementary.pdf]

Supplementary for:

Genome-wide analyses of the temperature-responsive genetic loci of the pectinolytic plant pathogenic *Pectobacterium atrosepticum*

Natalia Kaczynska [1], Ewa Lojkowska [1], Magdalena Narajczyk [2] and Robert Czajkowski [3] *

[1] Laboratory of Plant Protection and Biotechnology, Intercollegiate Faculty of Biotechnology, University of Gdansk and Medical University of Gdansk, Antoniego, Abrahama 58, 80-307 Gdansk, Poland; natalia.kaczynska@ug.edu.pl (N.K.); ewa.lojkowska@ug.edu.pl (E.L.)
[2] Laboratory of Electron Microscopy, Faculty of Biology, University of Gdansk, Wita Stwosza 59, 80-308 Gdansk, Poland; magdalena.narajczyk@ug.edu.pl
[3] Laboratory of Biologically Active Compounds, Intercollegiate Faculty of Biotechnology, University of Gdansk and Medical University of Gdansk, Antoniego, Abrahama 58, 80-307 Gdansk, Poland
* Correspondence: robert.czajkowski@ug.edu.pl; Tel.: +48-58-5236333

Table S1. Characterization of *P. atrosepticum* SCRI1043 the wild-type strain and its transposon mutant strains based on PCWDEs activities and motility

No.	*P. atrosepticum*	PCWDEs [a]			Motility [b]	Biofilm formation [c]	Potato-tissue macerating ability [d]
		Pectinolytic enzymes	Cellulases	Proteases		OD_{595}	Macerated tissue (g)
1	SCRI1043	++	+	++	++	0.023 ± 0.0037	1,92 ± 0,86
2	PbaTn5-1	+++	+	++	++	0.021 ± 0.0053	2,08 ± 0,97
3	PbaTn5-4	+++	+	++	++	0.021 ± 0.0039	2,42 ± 1,11
4	PbaTn5-8	+++	+	++	++	0.021 ± 0.0070	2,08 ± 0,80
5	PbaTn5-11	+++	+	++	++	0.020 ± 0.0029	2,33 ± 0,75
6	PbaTn5-13	+++	+	++	++	0.021 ± 0.0041	2,17 ± 0,75
7	PbaTn5-14	+++	+	++	++	0.020 ± 0.0029	2,00 ± 0,32
8	PbaTn5-15	+++	+	++	++	0.025 ± 0.0039	1,92 ± 0,74
9	PbaTn5-16	+++	+	++	++	0.021 ± 0.0037	1,67 ± 0,68
10	PbaTn5-19	+++	+	++	++	0.021 ± 0.0027	2,67 ± 0,61
11	PbaTn5-23	+++	+	++	++	0.024 ± 0.0044	2,08 ± 0,86

No.	*P. atrosepticum*	PCWDEs [a]			Motility [b]	Biofilm formation [c]	Potato-tissue macerating ability [d]
		Pectinolytic enzymes	Cellulases	Proteases		OD_{595}	Macerated tissue (g)
12	PbaTn5-26	+++	+	++	++	0.024 ± 0.0031	1,92 ± 0,58
13	PbaTn5-29	+++	+	++	++	0.020 ± 0.0021	2,33 ± 0,75
14	PbaTn5-38	+++	+	++	++	0.021 ± 0.0070	1,92 ± 0,38
15	PbaTn5-43	+++	+	++	++	0.029 ± 0.0043 **	1,58 ± 1,16
16	PbaTn5-46	+++	+	++	++	0.025 ± 0.0055	2,08 ± 0,38
17	PbaTn5-48	+++	+	++	++	0.021 ± 0.0037	2,08 ± 0,86
18	PbaTn5-54	+++	+	++	++	0.023 ± 0.0045	2,08 ± 0,66
19	PbaTn5-55	+++	+	++	++	0.021 ± 0.0052	1,50 ± 0,63
20	PbaTn5-A2	+++	+	++	++	0.024 ± 0.0034	2,00 ± 1,00
21	PbaTn5-A6	+++	+	++	++	0.020 ± 0.0042	1,25 ± 0,52
22	PbaTn5-A7	+++	+	++	++	0.024 ± 0.0025	2,17 ± 0,88
23	PbaTn5-A21	+++	+	++	++	0.020 ± 0.0016	1,92 ± 0,38
24	PbaTn5-A27	+++	+	++	++	0.020 ± 0.0049	2,00 ± 0,77
25	PbaTn5-A29	+++	+	++	++	0.021 ± 0.0062	1,92 ± 0,86
26	PbaTn5-A33	+++	+	++	++	0.021 ± 0.0071	2,25 ± 0,82
27	PbaTn5-A43	+++	+	++	++	0.020 ± 0.0052	3,33 ± 0,93 *
28	PbaTn5-B9	+++	+	++	++	0.021 ± 0.0027	1,83 ± 0,75
29	PbaTn5-B16	+++	+	++	++	0.020 ± 0.0047	2,08 ± 0,58
30	PbaTn5-B25	+++	+	++	++	0.024 ± 0.0059	2,25 ± 0,52
31	PbaTn5-B30	+++	+	++	++	0.025 ± 0.0029	2,67 ± 0,88
32	PbaTn5-B31	+++	+	++	++	0.018 ± 0.0045 **	2,50 ± 0,63
33	PbaTn5-B36	+++	+	++	++	0.026 ± 0.0057	2,25 ± 0,69
34	PbaTn5-B42	+++	+	++	++	0.027 ± 0.0058	2,67 ± 0,52
35	PbaTn5-B52	+++	+	++	++	0.023 ± 0.0051	2,50 ± 0,95
36	PbaTn5-B53	+++	+	++	++	0.026 ± 0.0066	2,17 ± 0,52
37	PbaTn5-B58	+++	+	++	++	0.030 ± 0.0056 **	1,83 ± 0,61
38	PbaTn5-B63	+++	+	++	++	0.021 ± 0.0034	2,25 ± 0,76

No.	*P. atrosepticum*	PCWDEs [a]			Motility [b]	Biofilm formation [c]	Potato-tissue macerating ability [d]
		Pectinolytic enzymes	Cellulases	Proteases		OD_{595}	Macerated tissue (g)
39	PbaTn5-B76	+	+	-	+/-	0.036 ± 0.0061 ***	0,25 ± 0,42 **
40	PbaTn5-B78	+++	+	++	++	0.022 ± 0.0069	1,83 ± 0,41
41	PbaTn5-B80	+++	+	++	++	0.023 ± 0.0046	2,00 ± 0,55

[a] PCWDEs production:'-' no halo. '+' halo diameter 0.1-10 mm. '++' 11-15 mm. '+++': 16-30 mm.

[b] Motility: '-' colony diameter < 5 mm. '+' 5-15 mm. '++' 16-30 mm. '+++' 31-50 mm. '++++' > 50 mm.

[c] The biofilm formation was measured by determining the OD_{565} after staining with crystal violet. Each value represents the mean (±SD) of two independent experiments, each performed in duplicate. Asterisks indicate statistically significant differences in the biofilm formation of the mutants compared with the wild-type strain *P. atrosepticum* SCRI1043 (***, p-value < 0.001; **, p-value < 0.01; *, p-value < 0.05). Statistical significance was determined by Student's *t*-test.

[d] The weight (g) of macerated potato tuber tissue, measured after a 72 h infection of potato tuber slices. The values are expressed as the mean (± SD) of two biological replicates for n=6 potato tuber slices inoculated with each strain. Asterisks indicate statistically significant differences in the degree of maceration of the mutants compared with the wild-type strain *P. atrosepticum* SCRI1043 (***, p-value < 0.001; **, p-value < 0.01; *, p-value < 0.05). Statistical significance was determined by Student's *t*-test.

A

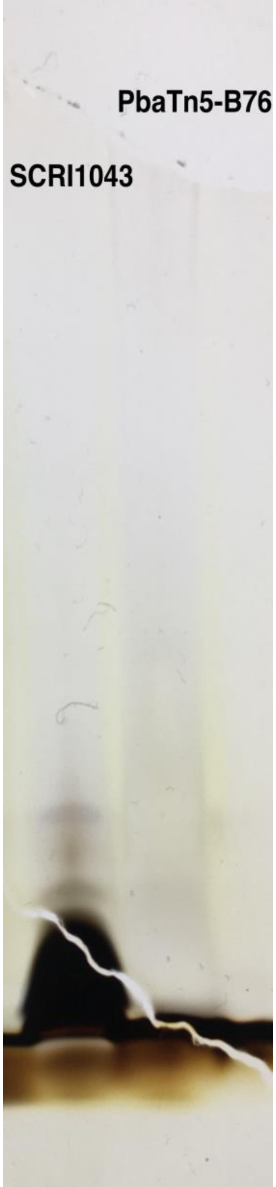

B

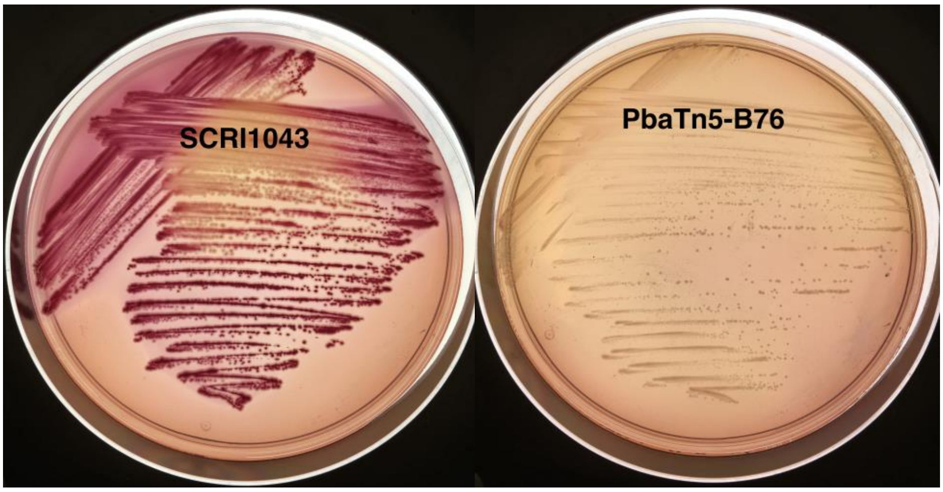

Figure S1. LPS profiles obtained by SDS-PAGE analysis and silver staining of crude LPS from the *P. atrosepticum* wild-type strain SCRI1043 and transposon mutant PbaTn5-B76. Crude LPS was extracted from the bacterial suspension with a concentration of 5 McFarland **(A)**. Phenotypes of the *P. atrosepticum* wild-type strain SCRI1043 and transposon mutant PbaTn5-B76 on MacConkey agar plates **(B).**
